# The Association of Elevated Factor VIII and von Willebrand Factor (vWF) Levels with SYNTAX Score in Patients with Chronic Coronary Syndrome

**DOI:** 10.3390/biomedicines13092284

**Published:** 2025-09-17

**Authors:** Predrag Djuric, Zorica Mladenovic, Zoran Jovic, Snjezana Vukotic, Marijan Spasic, Mirjana Mijuskovic, Brankica Terzic, Zoran Radojicic, Nina Radisavljevic, Marko Djuric, Dragan Djuric

**Affiliations:** 1 Clinic for Cardiology, Military Medical Academy, 11040 Belgrade, Serbia; zoz3377@gmail.com (Z.M.); zockydr@gmail.com (Z.J.); 2 Clinic for Urgent Internal Medicine, Military Medical Academy, 11040 Belgrade, Serbia; svukotic.luka@gmail.com (S.V.); marijans74@gmail.com (M.S.); 3 Clinic for Nephrology, Military Medical Academy, 11040 Belgrade, Serbia; mirjana.mijuskovic@gmail.com (M.M.); brankica.terzic@gmail.com (B.T.); 4 Faculty of Organizational Sciences, University of Belgrade, 11000 Belgrade, Serbia; zoran.radojicic@fon.bg.ac.rs; 5 Institute of Medical Physiology “Richard Burian”, Faculty of Medicine, University of Belgrade, 11000 Belgrade, Serbia; nina_radisavljevic@outlook.com (N.R.); dragan.djuric@med.bg.ac.rs (D.D.); 6 Clinic for Anesthesiology and Intensive Care, University Clinical Hospital Center “Dr Dragisa Misovic-Dedinje”, 11040 Belgrade, Serbia; drdjuric89@hotmail.com; 7 Department of Anesthesiology, Reanimatology and Intensive Care, Faculty of Medicine, University of Belgrade, 11000 Belgrade, Serbia

**Keywords:** chronic coronary syndrome, factor VIII, SYNTAX score, von Willebrand factor

## Abstract

**Background and Objectives:** Factor VIII (FVIII) and the von Willebrand factor (vWF) are key components of hemostatic balance. Disruption of the vWF-ADAMTS13 axis, characterized by elevated vWF and reduced ADAMTS13 activity has been implicated in thrombotic disorders, including COVID-19-asscoiated coagulopathy, where this imbalance correlates with disease severity and mortality. This study evaluated the relationship between plasma FVIII and vWF levels and the severity of coronary artery disease (CAD), as assessed by the SYNTAX score. **Methods:** We enrolled 82 patients with chronic coronary syndrome (CCS) and a positive treadmill test who underwent elective coronary angiography. Based on the SYNTAX score, patients were divided into three groups: Group I (≤22), Group II (23–32), and Group III (≥33). **Results:** FVIII levels varied significantly (Group I: 2.25 ± 0.75; Group III: 2.97 ± 0.95; *p* = 0.007), with an OR of 3.632 (95% CI: 1.116–11.826; *p* = 0.03). vWF levels differed significantly across SYNTAX groups (Group I: 1.16 ± 0.59; Group II: 1.52 ± 0.62; Group III: 1.49 ± 0.80; *p* = 0.040). vWF > 1.75 was more frequent in Groups II and III, with an odds ratio (OR) of 4.909 (95% CI: 1.429–16.864; *p* = 0.01) for Group III vs. Group I. Fibrinogen and C-reactive protein (CRP) were elevated in patients with SYNTAX scores ≥33. In multinomial logistic regression analysis, FVIII emerged as the sole independent predictor of CAD complexity (*p* = 0.004), while the vWF showed significance in pairwise comparison (Group II vs. Group I; OR = 3.433, *p* = 0.049). **Conclusions:** This study demonstrated significant differences in hemostatic and inflammatory biomarkers across SYNTAX score categories reflecting CAD severity in CCS patients. FVIII emerged as an independent predictor of CAD complexity, while the vWF demonstrated significant associations in specific subgroup comparisons. The observed vWF-ADAMTS13 axis dysregulation supports the rationale for investigating vWF-targeted therapeutics, including agents such as caplacizumab, in cardiovascular disease management. These findings require validation in larger studies.

## 1. Introduction

Chronic coronary syndrome (CCS) encompasses a spectrum of clinical presentations resulting from structural and functional abnormalities in the coronary arteries and/or microcirculation. It is most commonly caused by the atherosclerotic obstruction of epicardial coronary arteries, although microvascular disease may also contribute in certain cases. Patients with coronary artery disease (CAD) typically present with symptoms of CCS, with atherosclerosis forming the underlying pathophysiological substrate. Numerous factors, including markers of hemostasis, thrombosis, and inflammation, are involved in the initiation and progression of this complex vascular process [1,2,3,4].

Factor VIII (FVIII) is primarily synthesized by endothelial cells, and its elevated plasma levels have been observed in individuals with obesity, diabetes mellitus (DM), and a range of chronic inflammatory conditions [5]. The functional interplay between FVIII and the Von Willebrand factor (vWF) is critical: the vWF acts as a carrier protein, shielding FVIII from proteolytic inactivation by activated protein C and protein S, while also directing it to sites of endothelial injury [6]. Although concomitant elevations of the vWF and FVIII have been reported in acute coronary syndrome (ACS), corresponding evidence in CCS remains scarce. Large-scale prospective studies have identified elevated FVIII as a potential predictor of coronary events and stroke, but the independence of this association from traditional cardiovascular risk factors requires clarification [7,8].

The vWF is a glycoprotein primarily secreted by vascular endothelial cells and released in response to endothelial injury, making it a widely used marker of endothelial dysfunction [9,10]. The vWF plays a pivotal role in platelet adhesion and aggregation, particularly under high shear stress conditions, such as those found in stenotic coronary arteries with vulnerable plaques [11,12]. In ACSs, the vWF adheres to exposed subendothelial surfaces following plaque rupture, promoting platelet activation and thrombus formation [13]. While the association between elevated vWF levels and ACS is well established, its association with CAD severity in the context of CCS remains insufficiently studied [14].

The balance between the vWF and its cleaving protease ADAMTS13 is critical for maintaining microvascular homeostasis. Evidence from thromboinflammatory conditions, including COVID-19, indicates that elevated vWF levels combined with reduced ADAMTS13 activity are associated with disease severity and thrombotic risk. This highlights the potential clinical significance of the vWF-ADAMTS13 axis in cardiovascular pathology [15].

Fibrinogen has been linked to both the presence and severity of CAD, with studies demonstrating associations between elevated levels and adverse clinical outcomes [16,17,18,19]. More recent evidence has further linked increased fibrinogen concentrations with atherosclerotic plaque progression in both ACS and stable angina pectoris [20,21,22].

C-reactive protein (CRP) is a marker of systemic inflammation. While elevated CRP levels predict complex coronary lesions in ACS patients [23], their relationship with CAD severity in CCS remains inconclusive [24,25,26,27].

The severity of CAD can be objectively quantified using the SYNTAX score, a validated angiographic tool that assesses the extent and complexity of coronary lesions with ≥50% diameter stenosis in vessels measuring ≥1.5 mm [28]. The SYNTAX score provides important guidance in choosing optimal revascularization strategies: patients with a low SYNTAX score are generally best managed with percutaneous coronary intervention (PCI), those with an intermediate score may benefit from either PCI or coronary artery bypass grafting (CABG), while patients with a high SYNTAX score typically have superior outcomes with CABG.

The SYNTAX score was selected in this study as it represents a robust and widely validated angiographic tool that not only quantifies the anatomical complexity of CAD but also guides therapeutic decision-making, thereby providing a clinically meaningful measure of disease burden. Despite the numerous investigations addressing FVIII and the vWF in ACS, data on their association with the SYNTAX score and their prognostic value in CCS remain limited. This gap provides the rationale and novelty of the present study.

The aim of this study was to examine whether plasma levels of FVIII and the vWF are associated with the severity of CAD, as assessed by the SYNTAX score, and to determine whether these hemostatic factors independently predict coronary complexity in multinomial logistic regression analysis. This clinically oriented investigation progresses from patient characterization through biomarker analysis to predictive modeling, targeting specialists in cardiovascular and translational medicine.

## 2. Materials and Methods

### 2.1. Study Design and Participants

This cross-sectional observational study was conducted at the Clinic for Cardiology, Military Medical Academy, Belgrade, Serbia, between December 2017 and March 2018. It was carried out in accordance with the principles of the Declaration of Helsinki and was approved by the institutional review board of the Military Medical Academy, Belgrade, Serbia (Approval No. 3335-8, date 1 December 2017).

This study included 82 patients aged 18 to 80 years presenting with symptoms of CCS consistent with stable angina pectoris, such as chest pain and exercise intolerance. All patients had a positive treadmill exercise test and underwent elective coronary angiography. The duration of symptoms prior to diagnosis was approximately one year. Patients were consecutively enrolled after providing written informed consent. Based on coronary angiographic findings and the SYNTAX score, they were categorized into three groups: Group I (≤22 points), Group II (23–32 points), and Group III (≥33 points), in accordance with established clinical guidelines.

Exclusion criteria included the following: (1) the presence of ACS, (2) previous myocardial infarction, (3) history of PCI or surgical revascularization, (4) severe valvular disease, (5) active or chronic inflammatory, infectious, or malignant diseases, (6) autoimmune diseases requiring immunosuppressive or corticosteroid therapy, (7) thyroid dysfunction, (8) advanced renal or hepatic impairment, (9) metabolic disorders such as familial hyperlipoproteinemia, (10) endocrine disorders including pheochromocytoma and Cushing’s syndrome, (11) uncontrolled arterial hypertension (blood pressure >150/90 mmHg), (12) cognitive impairment that precluded reliable data collection, (13) pregnant women, and (14) those who declined to provide informed consent. Only patients who met all the inclusion and none of the exclusion criteria were included in the final analysis, in order to reduce potential confounding factors and ensure the internal validity of the study.

### 2.2. Data Collection and Assessment

In addition to FVIII and the vWF, standard biochemical parameters were measured, including fasting glucose, serum creatinine, total and low-density lipoprotein cholesterol (LDL), triglycerides, erythrocyte sedimentation rate (ESR), leukocyte count, CRP, and fibrinogen.

Whole blood samples were collected within 24 h of hospital admission and prior to coronary angiography. Venous blood was drawn from the antecubital vein into 3.2% sodium citrate tubes (Greiner Bio-One; Vacuette 9NC) from all patients in a fasting state. Samples were centrifuged at 1500 rpm for 15 min, and the supernatant was maintained at +15 to +25 °C until analysis.

FVIII levels were determined using the coagulometric method. A mixture of FVIII-deficient plasma and patient plasma was analyzed using an activated partial thromboplastin time (APTT)-based assay. Results were expressed as a percentage of pooled normal plasma, with the normal reference range of 0.7 to 1.5 (70–150%). Sample stability was up to 3 h.

vWF ristocetin cofactor activity was measured using the BC von Willebrand reagent on a Siemens BCS XP coagulation analyzer. This assay evaluates the ability of the vWF to agglutinate platelets in the presence of ristocetin. Results were expressed as a percentage of the mean plasma concentration in a healthy population, with the normal range defined as 0.58 to 1.72 (58–172%). Sample stability was up to 6 h.

Medication use was documented upon hospital admission. The following drug classes were included: statins, fibrates, antiplatelet agents (including thienopyridines), ACE inhibitors, insulin, oral hypoglycemic agents, diuretics, and nitrates.

### 2.3. Statistical Analysis

Statistical analysis was performed using SPSS software version 25.0. Mean values with standard deviation were reported for data with normal distribution, while median values with interquartile range (IQR) were used for data without normal distribution. Differences between groups were assessed using the Mann–Whitney test for comparisons between two independent groups and the Kruskal–Wallis test for multiple group comparisons. Categorical variables were compared using the Chi-square test (χ^2^).

Associations between FVIII, the vWF, and CAD severity (based on SYNTAX score categories) were further evaluated using univariate logistic regression analysis. Odds ratios (ORs) with 95% confidence intervals (CIs) were calculated using the Mantel–Haenszel method. Cluster analysis with Ward’s method was employed to determine optimal cut-off values for biomarker levels [29]. A *p* value of <0.05 was considered statistically significant.

Multinomial logistic regression analysis was performed to identify independent predictors of the SYNTAX score, adjusting for potential confounders including age, sex, DM, creatinine levels, FVIII, and the vWF. Model assumptions were verified, and statistical significance was set at a *p* value of <0.05.

## 3. Results

### 3.1. Demographic and Clinical Characteristics

Among the 82 participants with CCS included in the study, the average age was 65 ± 8 years, and 71.1% were male. The distribution of patients across SYNTAX score groups was as follows: 42 patients (51.2%) in group I (SYNTAX score ≤22), 20 patients (24.4%) in group II (SYNTAX score 23–32), and 20 patients (24.4%) in group III (SYNTAX score ≥33).

Demographic and clinical characteristics according to SYNTAX score categories are presented in Table 1. Statistically significant differences were observed among the three groups in terms of smoking status, physical activity, serum creatinine levels, and diastolic blood pressure at admission (*p* < 0.05). No significant differences were noted regarding age, sex, history of hypertension, family history of cardiovascular disease, DM, or systolic blood pressure.

Medication use did not differ significantly among the groups for any drug class analyzed, including statins, fibrates, thienopyridines, ACE inhibitors, insulin, oral hypoglycemic agents, diuretics, and nitrates (*p* > 0.05).

### 3.2. Laboratory Parameters

Biochemical parameters across groups are presented in Table 2. Among these parameters, only serum creatinine levels differed significantly between groups (*p* = 0.031).

Hemostatic and inflammatory markers are summarized in Table 3. We found significant differences among the groups regarding FVIII (*p* = 0.007), vWF (*p* = 0.040), fibrinogen (*p* = 0.030), and CRP levels (*p* = 0.017). No significant differences were observed for leukocyte count, estimated sedimentation rate (ESR), IL-6, D-dimer, plasminogen activator inhibitor-1 (PAI-1), prothrombin time or activated thromboplastin time.

### 3.3. Imaging and Angiographic Parameters

While echocardiographic parameters did not differ significantly among the groups, angiographic findings demonstrated statistically significant variations (Table 4). In particular, the number of affected and treated coronary arteries, as well as the involvement of the left anterior descending artery and the right coronary artery, differed significantly among the groups.

### 3.4. Hemostatic and Inflammatory Parameters According to SYNTAX Score Groups

**Figure 1:**
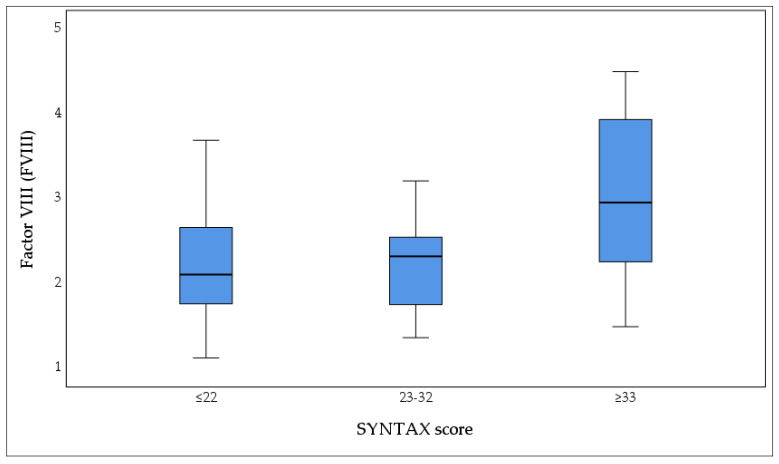
Plasma concentrations of coagulation factor VIII (FVIII) according to coronary artery disease severity, categorized by SYNTAX score: low (≤22, *n* = 42), intermediate (23–32, *n* = 20), and high (≥33, *n* = 20). The box plot demonstrates a progressive increase in FVIII levels with increasing CAD complexity, with the highest median FVIII concentrations observed in patients with high SYNTAX scores (≥33). Boxes represent the interquartile range (IQR), horizontal lines indicate median values, and whiskers denote the minimum and maximum values. Statistical significance was assessed using the Kruskal–Wallis test (*p* = 0.009). FVIII: factor VIII.

We detected significant differences between plasma levels of FVIII and severity of CAD according to SYNTAX score (Group I: 2.25 ± 0.75, Group II: 2.21 ± 0.53, Group III: 2.97 ± 0.95; *p* = 0.007) (Figure 1). The highest mean FVIII level was observed in Group III (2.97 ± 0.95).

**Figure 2:**
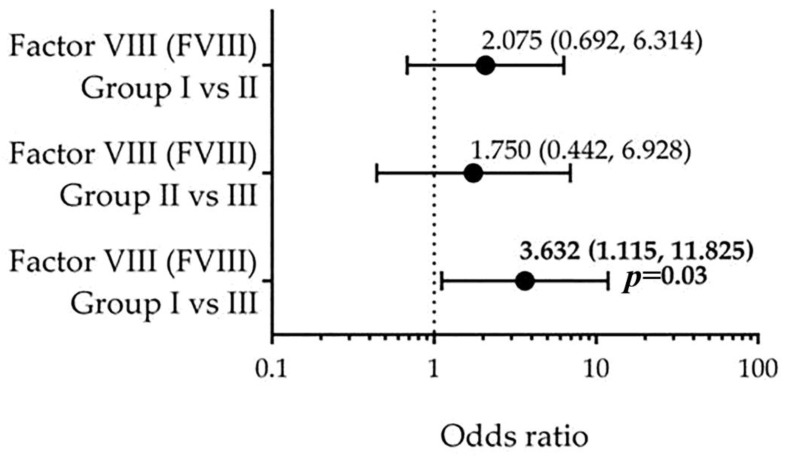
Odds ratios (OR) with 95% confidence intervals (CI) for severe coronary artery disease according to factor VIII (FVIII) levels, dichotomized at >2.5 vs. <2.5. Group I: SYNTAX score ≤22 (*n* = 42); Group II: SYNTAX score 23–32 (*n* = 20); Group III: SYNTAX score ≥33 (*n* = 20). Elevated FVIII levels (>2.5) were significantly associated with high CAD complexity when comparing Group I vs. Group III (OR = 3.632, 95% CI: 1.115–11.825, *p* = 0.03). No statistically significant associations were observed for Group I vs. II (OR = 2.075, 95% CI: 0.692–6.314) or Group II vs. III (OR = 1.750, 95% CI: 0.442–6.928) comparisons. Odds ratios were calculated using the Mantel–Haenszel method. Statistical significance was defined as *p* < 0.05. FVIII: factor VIII.

We subsequently estimated the OR for severe CAD based on dichotomized FVIII levels (>2.5 vs. ≤2.5) using the Mantel–Haenszel method. An unadjusted OR of 3.632 (95% CI: 1.115–11.825; *p* = 0.03) was observed for patients with FVIII levels >2.5 (Figure 2).

**Figure 3:**
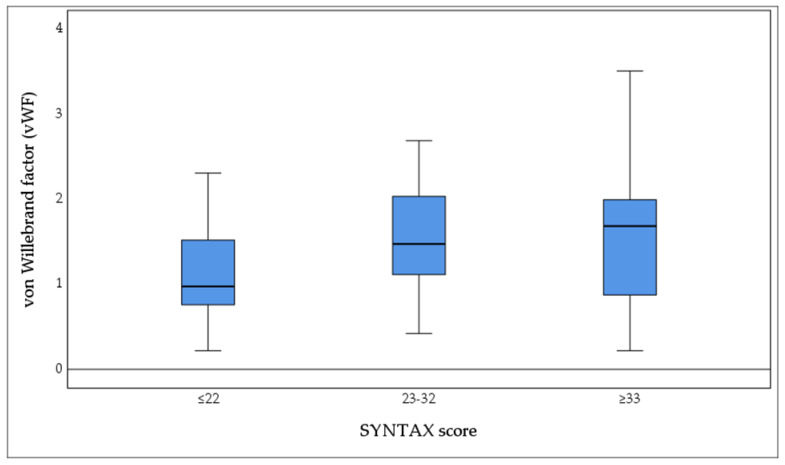
Plasma concentrations of the von Willebrand factor (vWF) according to coronary artery disease severity, categorized by SYNTAX score: low (≤22, *n* = 42), intermediate (23–32, *n* = 20), and high (≥33, *n* = 20). Boxes represent the interquartile range (IQR), horizontal lines indicate median values, and whiskers denote the minimum and maximum values. Statistical significance was assessed using the Kruskal–Wallis test (*p* = 0.040), indicating significant differences in vWF levels across the three complexity groups. vWF: von Willebrand factor.

Plasma levels of the vWF differed significantly across SYNTAX score groups (Group I: 1.16 ± 0.59, Group II: 1.52 ± 0.62, Group III: 1.49 ± 0.80, Kruskal–Wallis *p* = 0.040) (Figure 3).

**Figure 4:**
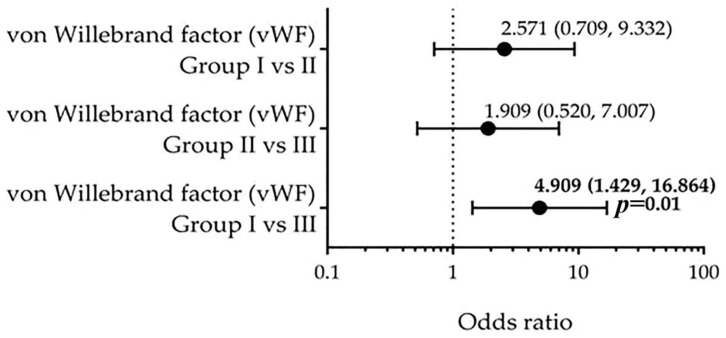
Odds ratios (OR) with 95% confidence intervals (CI) for severe coronary artery disease according to von Willebrand factor (vWF) levels, dichotomized at >1.75 vs. ≤1.75. Group I: SYNTAX score ≤22 (*n* = 42); Group II: SYNTAX score 23–32 (*n* = 20); Group III: SYNTAX score ≥33 (*n* = 20). Elevated vWF levels were significantly associated with high CAD complexity when comparing Group I vs. Group III (OR = 4.909, 95% CI: 1.429–16.864, *p* = 0.01). No statistically significant associations were observed for Group I vs. II (OR = 2.571, 95% CI: 0.709–9.332) or Group II vs. III (OR = 1.909, 95% CI: 0.520–7.007). Odds ratios were calculated using the Mantel–Haenszel method. Statistical significance was defined as *p* < 0.05. vWF: von Willebrand factor.

The vWF was significantly higher in groups II and III compared to group I (SYNTAX score: *p* = 0.023 and 0.071, respectively). We then estimated the OR for severe CAD according to dichotomized vWF (>1.75 vs. ≤1.75) using the Mantel–Haenszel method. Patients with vWF >1.75 had a 4.909-fold greater odds of severe CAD (OR 4.909, 95% CI 1.429–16.864; *p* = 0.01) (Figure 4).

**Figure 5:**
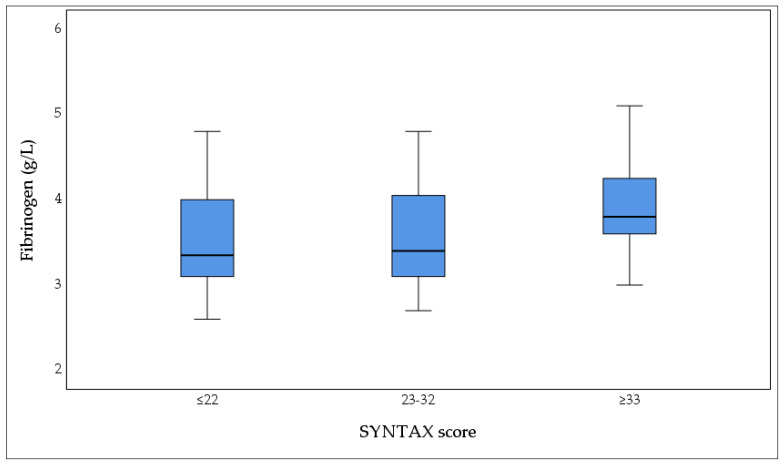
Plasma concentrations of fibrinogen (g/L) according to the severity of coronary artery disease, categorized by SYNTAX score: low (≤22, *n* = 42), intermediate (23–32, *n* = 20), and high (≥33, *n* = 20). The box plot demonstrates a progressive increase in fibrinogen levels with increasing CAD complexity, with the highest median fibrinogen concentrations observed in patients with high SYNTAX scores (≥33). Boxes represent the interquartile range (IQR), horizontal lines indicate median values, and whiskers denote the minimum and maximum values. Statistical significance was assessed using the Kruskal–Wallis test (*p* = 0.030).

Fibrinogen values showed statistically significant differences between groups according to the SYNTAX score (Kruskal–Wallis test, *p* = 0.030). The results are shown as mean value ± standard deviation (X ± SD): Group I with a low SYNTAX score ≤22 (3.53 ± 0.107 g/L), Group II with SYNTAX score 23–32 (3.59 ± 0.144 g/L), and Group III with SYNTAX score ≥33 (3.93 ± 0.122 g/L), as shown in Figure 5.

**Figure 6:**
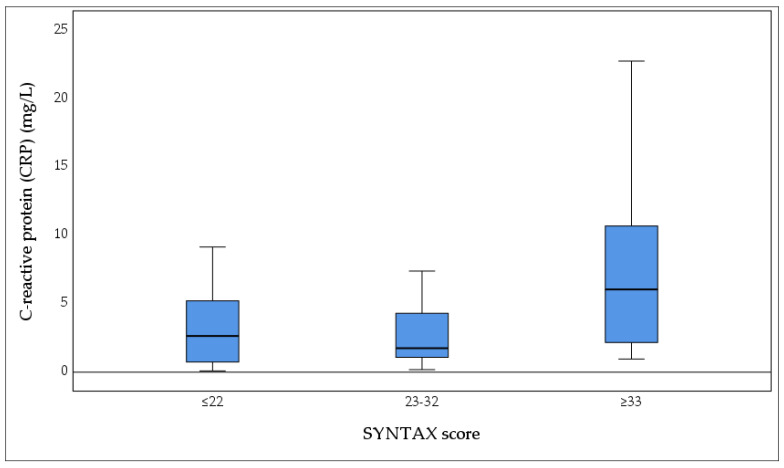
Plasma concentrations of C-reactive protein (CRP, mg/L) according to the severity of coronary artery disease, categorized by SYNTAX score: low (≤22 points, *n* = 42), intermediate (23–32 points, *n* = 20), and high (≥33 points, *n* = 20). The box plot demonstrates markedly elevated CRP levels in patients with increasing CAD complexity, with the highest median fibrinogen concentrations observed in patients with high CAD complexity compared to low and intermediate complexity groups. Boxes represent the interquartile range (IQR), horizontal lines indicate median values, and whiskers denote the minimum and maximum values. Statistical significance was assessed using the Kruskal–Wallis test (*p* = 0.017). CRP: C-reactive protein.

Analysis of CRP values demonstrated statistically significant differences among the groups (Kruskal–Wallis test, *p* = 0.017), with the most prominent difference observed between Group III and Group I (Figure 6). The highest CRP levels were recorded in Group III (SYNTAX score ≥33).

### 3.5. Identification of Independent Predictors of Coronary Artery Disease Severity According to SYNTAX Score

To further explore factors independently associated with the severity of CAD, a multinomial logistic regression analysis was performed, with CAD severity categorized according to SYNTAX score serving as the dependent variable (Table 5). The model included age, sex, presence of DM, creatinine clearance, FVIII and vWF levels as covariates. Among these variables, only FVIII emerged as a statistically significant independent predictor (*p* = 0.004), whereas the vWF did not retain significance in the adjusted model (*p* = 0.115), indicating that its association observed in univariate analysis may have been confounded by other covariates.

The multinomial logistic regression model demonstrated satisfactory statistical validity and overall model fit. The model significantly outperformed the null model (χ^2^ = 27.297, *p* = 0.018), and goodness-of-fit statistics (Pearson *p* = 0.290; Deviance *p* = 0.649) indicated an adequate alignment between the observed and predicted data. Pseudo-R-squared values, particularly Nagelkerke’s *R*^2^ = 0.328, suggested a moderate degree of explanatory power.

Among the variables included, FVIII was identified as the strongest predictor of CAD severity (*p* = 0.004), demonstrating a significant association with higher SYNTAX scores. Specifically, elevated FVIII levels were associated with 2.8-fold increased odds of classification into Group III, compared to Group I (OR = 2.832, *p* = 0.041). While the vWF did not achieve statistical significance in the overall model (*p* = 0.115), it showed a significant association in the pairwise comparison between Group II and Group I, with higher levels linked to a threefold increase in the odds of being categorized within the intermediate SYNTAX score group (OR = 3.433, *p* = 0.049). The remaining covariates, including age, sex, and creatinine clearance were not independently associated with CAD severity, although creatinine clearance approached borderline significance (*p* = 0.075). These findings underscore the potential clinical relevance of hemostatic biomarkers, particularly FVIII, and to a lesser extent the vWF, in stratifying the anatomical complexity of CAD, as assessed by the SYNTAX score.

## 4. Discussion

In this study, we investigated potential associations between plasma levels of FVIII and the vWF and the angiographic severity of CAD, as assessed by the SYNTAX score, in patients with CCS. Our findings demonstrated significant differences in biomarker levels across SYNTAX score categories. In multivariable analysis, FVIII emerged as the most robust independent predictor of CAD complexity, whereas the vWF was significantly associated with disease severity in univariate analysis, but did not retain statistical significance after adjustment for potential confounding factors.

FVIII is a well-recognized acute-phase reactant with a central role in hemostasis. As a key cofactor in the coagulation cascade, it accelerates the activation of Factor X and subsequent thrombin generation, thereby amplifying thrombus formation [30]. Approximately 11% of the adult population worldwide present with elevated FVIII levels [31], with particularly high concentrations observed in conditions such as myocardial infarction, postoperative states, pregnancy, malignancy, liver disease, hyperthyroidism, chronic kidney disease, and both arterial and venous thromboembolism [32]. FVIII also shows strong correlations with traditional cardiovascular risk factors, including advanced age, DM, increased body mass, and several prothrombotic and inflammatory biomarkers [33].

Elevated FVIII levels have been documented in various clinical presentations of ACS, regardless of traditional risk factors [34]. Studies demonstrated elevated FVIII activity in both myocardial infarction and non-obstructive coronary arteries (MINOCA) and obstructive disease (MICAD), suggesting that increased FVIII may be a consistent feature across the ACS spectrum [35]. Elevated FVIII activity has also been observed in 72.40% of patients with cryptogenic ischemic stroke, and was associated with a higher incidence of in-hospital thrombotic events [36]. These data collectively suggest that FVIII may represent a shared mediator of arterial thrombosis across vascular territories, independent of established risk factors.

In our study, we observed that elevated FVIII plasma levels differed significantly across SYNTAX score groups. Notably, patients with FVIII concentrations exceeding 2.5 had 3.6-fold higher odds of being classified in the subgroup with a SYNTAX score ≥33.

Although the vWF did not emerge as an independent predictor of CAD complexity in our multinomial logistic regression analysis, its univariate association with disease severity aligns with previous research implicating the vWF in atherothrombotic processes. While direct evaluations of vWF levels in relation to SYNTAX score remain scarce, several studies have reported a positive correlation between elevated vWF concentrations and increased risk of CAD [37,38]. A comprehensive meta-analysis of prospective studies confirmed an increased incidence of CAD in individuals with high vWF levels in the general population [39]. More recently, Fan et al. conducted a systematic review, demonstrating that plasma vWF levels measured at 24 h and 48 h after admission were significantly higher in patients who developed major adverse cardiovascular events compared to those who did not [40]. Kaikita et al. [41] observed significantly elevated vWF antigen levels in patients with acute myocardial infarction (AMI) compared to those with exertional angina, with an inverse correlation with ADAMTS-13, suggesting a pathological imbalance that favors thrombosis. The pronounced vWF elevation in ACS was further supported by a large-scale study in ST-elevation myocardial infarction (STEMI) patients, where vWF concentrations were 1.5 times higher compared to controls, reinforcing its role in plaque rupture and thrombosis [42]. Moreover, a recent case–control study showed that the combination of high vWF antigen (>150%) and low ADAMTS13 levels (≤0.64 µg/mL) was associated with a fourfold increased risk of thrombotic events, independent of age, gender, blood group, and CRP levels [43]. Collectively, these data suggest that assessing the entire vWF-ADAMTS13 axis may offer greater clinical insight than measuring the vWF alone.

Parvathareddy et al. [44] found significantly elevated vWF antigen levels in patients with more severe CAD, as determined by the modified Gensini score. In contrast, Leiva et al. [45] explored outcomes in 136 patients with von Willebrand disease (VWD) following AMI and reported a higher rate of bleeding-related readmissions but no significant increase in arterial thrombosis, suggesting that VWD may confer a protective effect against thrombotic complications. Consistently, Mihyawi and colleagues observed a lower prevalence and risk of CAD in VWD patients compared to controls, providing epidemiological support for the pathogenic role of the vWF in coronary atherosclerosis [46].

Advanced intravascular imaging techniques, such as intravascular ultrasound (IVUS), have provided valuable mechanistic insights into the link between the vWF and coronary atherosclerosis. In a study involving 91 patients undergoing PCI, Kato et al. [47] reported a significant association between elevated vWF levels and increased plaque burden, quantified as percent atheroma volume (PAV). Importantly, this association remained significant even among patients receiving statin therapy, suggesting that the vWF may reflect residual cardiovascular risk beyond lipid control. Similarly, Sonneveld et al. [48] demonstrated a positive correlation between vWF levels and both the presence and extent of atherosclerotic plaques using IVUS in CAD patients. These findings support the role of the vWF not only as a biomarker of clinical outcomes but also as an indicator of the anatomical burden of coronary atherosclerosis.

The biological plausibility of our findings is further supported by the dual role of the vWF in hemostasis and inflammation. The vWF promotes platelet adhesion and aggregation under high shear stress conditions found in stenotic coronary arteries [49] and facilitates leukocyte recruitment at sites of vascular injury, thereby amplifying local inflammation [50]. Together, these actions contribute to both atherogenesis and plaque destabilization.

While vWF elevation may initially reflect endothelial dysfunction and injury, emerging evidence suggests it also plays an active causal role in inflammatory processes. The vWF can serve as a scaffold for neutrophil extracellular traps (NETs), which form upon vascular injury and consist of DNA fibers with citrullinated histones that enhance prothrombotic capacity. NETs colocalize with the vWF in venous thrombi and can catch adhesive plasma proteins, including the vWF, further enhancing platelet recruitment. This interaction through NETs binding to the A1 domain of the vWF demonstrates that the vWF functions beyond a passive biomarker to actively participate in inflammatory thrombosis [51,52].

The role of the vWF in thrombosis differs across vascular beds. In the arterial circulation, its prothrombotic effect is mainly related to platelet adhesion under high shear stress, whereas in venous thrombosis the association was long thought to be indirect, mediated primarily through its function as a carrier of FVIII. More recent clinical and experimental studies, however, suggest that the vWF itself can also directly contribute to the formation of venous thrombi [51,53].

Beyond these functional roles, plasma vWF levels are strongly influenced by genetic regulatory mechanisms. Up to 60% of the vWF variability is genetically determined, with contributions from vWF gene polymorphisms, quantitative trait loci, and ABO blood group status, while epigenetic mechanisms, environmental exposures such as smoking, and biological factors like aging further modulate expression [54]. In parallel, inflammatory cytokines including IL-6, IL-8, and TNF-α stimulate endothelial release of ultra-large vWF multimers and impair their cleavage by ADAMTS13, leading to an accumulation of prothrombotic forms in the circulation. This interplay between genetic predisposition and inflammatory signaling provides a mechanistic explanation for the observed variability in vWF levels and their role in thrombosis [55].

Mechanistically, elevated vWF levels in CAD likely mirror endothelial dysfunction. In atherosclerotic vessels, reduced endothelial nitric-oxide-synthase (eNOS) activity diminishes nitric-oxide (NO) bioavailability, relieving the inhibition of vWF release from endothelial cells [56,57]. Because NO is a physiological inhibitor of vWF secretion [58], the disruption of the NO–vWF regulatory axis fosters a prothrombotic and pro-inflammatory milieu.

Our results support the view that circulating the vWF reflects underlying endothelial dysfunction, a critical process in atherosclerosis progression [59,60,61]. While the vWF showed a significant univariate correlation with the SYNTAX score in our study, multinomial logistic regression analysis revealed that the vWF did not retain an independent predictive value.

To our knowledge, no prior study has specifically examined the relationship between FVIII levels and CAD severity as quantified by the SYNTAX scoring system. However, large population-based studies demonstrated that elevated FVIII levels were independently associated with an increased risk of coronary heart disease, and to a lesser extent, stroke, even after adjustment for traditional cardiovascular risk factors and CRP [62,63,64,65].

The differential performance of hemostatic biomarkers in our multinomial logistic regression offers valuable insights into their relative clinical utility. Although both FVIII and the vWF were elevated in patients with more complex CAD, as assessed by the SYNTAX score, only FVIII retained independent predictive value after adjustment for potential confounders. This finding is particularly notable given the well-established biological interdependence between these two factors, suggesting that FVIII may serve as a more reliable biomarker for quantifying coronary atherosclerosis burden. The independent predictive capacity of FVIII—beyond traditional cardiovascular risk factors and vWF levels—highlights its potential role in the risk stratification of patients with advanced CAD in clinical settings.

Beyond the hemostatic factors discussed, our study revealed significant differences in inflammatory biomarkers across different CAD severity groups. Patients with SYNTAX scores ≥33 exhibited significantly elevated levels of fibrinogen and CRP compared to those with lower scores, consistent with previous research demonstrating associations between elevated inflammatory biomarkers and higher clinical SYNTAX scores in stable CAD [66].

The concomitant elevation of both hemostatic (FVIII, vWF) and inflammatory (fibrinogen, CRP) markers observed in patients with a SYNTAX score ≥33 suggests a complex interplay between thrombosis and inflammation in atherosclerotic disease. CRP serves as a valuable biomarker of systemic inflammation with established prognostic significance in CVD, with research demonstrating associations between elevated CRP levels and increased risk of major adverse cardiovascular events [67,68]. Similarly, fibrinogen functions at the intersection of inflammatory and thrombotic pathways in CAD, contributing to endothelial dysfunction and atherosclerotic plaque formation. Multiple clinical investigations have established correlations between elevated fibrinogen levels and adverse cardiovascular outcomes [69]. A large-scale meta-analysis encompassing over 150,000 subjects across multiple studies confirmed significant associations between fibrinogen concentrations and cardiovascular mortality [70].

Our findings have potential clinical and therapeutic implications. Several interventions targeting the vWF pathway have been investigated, though with varying results. While Revacept failed to demonstrate benefits in stable CAD patients [71], targeted thrombolysis approaches show promise [72]. Conventional anticoagulant and antiplatelet therapies may also modulate hemostatic factors [73,74]. Caplacizumab, a bivalent immunoglobulin that blocks the interaction between the vWF and GPIbα receptor, particularly under high shear stress conditions. Its clinical use in acquired thrombotic thrombocytopenic purpura (TTP) demonstrates the therapeutic potential of directly targeting the vWF-ADAMTS13 axis in thrombotic disorders [75].

The findings of this study demonstrate significant differences in plasma levels of FVIII and the vWF along with inflammatory markers such as fibrinogen and CRP, across different SYNTAX score categories in patients with CCS. Elevated FVIII levels, in combination with SYNTAX score assessment, may enhance the identification of patients at higher risk for complex coronary disease, potentially informing revascularization strategy selection between PCI and CABG in cases where anatomical complexity alone provides insufficient guidance for clinical decision-making. However, several important limitations that restrict the interpretation of these findings must be acknowledged. First, the relatively small sample size (*n* = 82) limits the statistical power and generalizability of the findings, particularly in subgroup analyses. Second, the observational, cross-sectional design precludes the establishment of causal relationships between elevated hemostatic and inflammatory markers and CAD severity. Although we performed multinomial logistic regression analysis to assess independent associations, the strength of evidence from this adjusted model is limited by our relatively small sample size, which may have insufficient statistical power to detect meaningful associations with traditional cardiovascular risk factors. Notably, none of the classical confounders (age, sex, DM, or creatinine levels) reached statistical significance in our model, likely reflecting the exploratory nature of this study rather than the true absence of associations. Therefore, while FVIII emerged as the sole independent predictor of SYNTAX score severity, these findings should be interpreted cautiously and require validation in larger, adequately powered cohorts. Third, the absence of longitudinal follow-up data restricts our ability to assess the prognostic value of the vWF and FVIII levels over time, such as their impact on clinical outcomes like myocardial infarction, revascularization, or mortality. Additionally, we did not adjust for several potential confounders, including statin use and other inflammatory conditions beyond CRP measurement.

Future research should aim to validate these findings in larger, multicenter cohorts encompassing diverse patient populations, to confirm the independent associations of FVIII and the vWF with the severity of CAD. Longitudinal studies are particularly needed to determine whether these biomarkers predict adverse cardiovascular outcomes over time, independent of established risk factors and whether their modulation through pharmacologic or lifestyle interventions could improve clinical prognosis. Additionally, interventional trials targeting the vWF/FVIII pathway may provide valuable insights into their potential as therapeutic targets in atherosclerotic disease. The development of more sensitive and specific assays for ultra-large vWF multimers could provide better risk stratification and monitoring capabilities. The investigation of novel vWF antagonists as therapeutic targets represents an emerging area with potential clinical applications. Moreover, exploring the role of the vWF and other coagulation factors in cancer-associated thrombosis and autoimmune diseases may reveal important mechanistic insights relevant to CAD pathophysiology.

## 5. Conclusions

Our study demonstrated significant differences in plasma levels of FVIII, the vWF, fibrinogen, and CRP across SYNTAX score categories reflecting CAD severity in patients with CCS. Patients with higher SYNTAX scores (≥33) exhibited notably elevated levels of these hemostatic and inflammatory biomarkers compared to those with lower scores. Among them, FVIII emerged as an independent predictor of CAD complexity in multinomial logistic regression analysis. Although the vWF did not retain statistical significance after adjustment, it showed a significant association in one contrast (Group II vs. I), where higher values were associated with more than a threefold increase in risk.

Given the limited sample size and the exploratory design of our study, these findings should be interpreted with caution. Additionally, our study did not employ systematic search methodology for literature review components of the discussion, which may have resulted in the selective citation of available evidence. Larger, more comprehensive studies are needed to confirm the independent association of FVIII with CAD severity and to further investigate the clinical relevance of both FVIII and the vWF in cardiovascular risk stratification. Despite losing statistical significance in the adjusted model, the observed elevation of the vWF in patients with more complex CAD may still reflect its pathophysiological involvement in atherothrombosis and warrants further investigation.

## Figures and Tables

**Table 1:** Demographic characteristics and clinical parameters of patients according to SYNTAX score.

Parameters		SYNTAX Score			
		Group I (≤22) (*n* = 42)	Group II (23–32) (*n* = 20)	Group III (≥33) (*n* = 20)	*p*
gender	female (%) male (%)	9 (21.40) 33 (78.60)	3 (15.00) 17 (85.00)	7 (35.00) 13 (65.00)	0.302 **
age (years)	median (IQR)	64.50 (10.75)	68.5 (15.25)	69.50 (11.50)	0.209 *
body mass index (kg/m^2^)	mean value (SD)	28.87 (3.94)	27.47 (3.19)	28.01 (2.99)	0.477 *
active smoking	*n* (%)	32 (76.20)	15 (75.00)	9 (45.00)	0.036 **
hypertension	*n* (%)	41 (97.60)	17 (85.00)	19 (95.00)	0.148 **
family history	*n* (%)	30 (71.40)	15 (75.00)	15 (75.00)	0.936 **
diabetes mellitus	*n* (%)	13 (31.00)	5 (25.00)	9 (45.00)	0.375 **
physical activity	*n* (%)	17 (40.50)	8 (40.00)	2 (10.00)	0.043 **
systolic blood pressure (mmHg)	mean value (SD)	138 (19)	134 (17)	139 (21)	0.782 *
diastolic blood pressure (mmHg)	mean value (SD)	83 (9)	84 (8)	75 (8)	0.004 *

*: *p* values obtained by the Kruskal–Wallis test; **: *p* values obtained by the Chi-square test. Statistically significant differences (*p* < 0.05) are given in bold. IQR: interquartile range; SD: standard deviation.

**Table 2:** Biochemical parameters according to SYNTAX score.

Parameters		SYNTAX Score			
		Group I (≤22) (*n* = 42)	Group II (23–32) (*n* = 20)	Group III (≥33) (*n* = 20)	*p* (Kruskal–Wallis Test)
fasting glucose (mmol/L)	mean value (SD)	6.31 (1.59)	5.84 (1.31)	7.37 (2.88)	0.094
	median (IQR)	6.00 (2.25)	5.50 (1.65)	6.55 (2.97)	
triglycerides (mmol/L)	mean value (SD)	2.01 (1.07)	1.03 (1.54)	1.54 (0.56)	0.247
	median (IQR)	1.74 (1.13)	1.44 (0.80)	1.59 (0.81)	
cholesterol (mmol/L)	mean value (SD)	5.28 (1.31)	4.94 (0.98)	4.81 (2.25)	0.373
	median (IQR)	5.25 (1.86)	4.73 (1.88)	4.93 (2.25)	
HDL cholesterol (mmol/L)	mean value (SD)	1.15 (0.26)	1.21 (0.30)	1.16 (0.25)	0.729
	median (IQR)	1.15 (0.33)	1.21 (0.37)	1.08 (0.26)	
LDL cholesterol (mmol/L)	mean value (SD)	3.21 (1.13)	2.84 (0.74)	3.03 (1.00)	0.429
	median (IQR)	3.21 (1.68)	2.69 (0.77)	3.05 (1.64)	
atherogenic index of plasma	mean value (SD)	0.21 (0.24)	0.14 (0.28)	0.11 (0.20)	0.305
	median (IQR)	0.21 (0.30)	0.17 (0.35)	0.12 (0.32)	
creatinine clearance (mL/min)	mean value (SD)	85.74 (16.98)	77.99 (18.72)	75.56 (21.84)	0.108
	median (IQR)	88.90 (18.15)	82.20 (27.60)	82.95 (29.87)	
creatinine (μmol/L)	mean value (SD)	80.79 (30.24)	87.60 (18.09)	89.55 (43.16)	**0.031**
	median (IQR)	73.50 (16.75)	87.50 (20.25)	83.00 (22.50)	
acidum uricum (umol/L)	mean value (SD)	344 (90)	240 (89)	364 (101)	0.526
	median (IQR)	340 (117)	319 (130)	361 (117)	
folic acid (nmol/L)	mean value (SD)	15.62 (8.93)	12.13 (11.07)	12.79 (8.39)	0.152
	median (IQR)	14.83 (12.86)	8.90 (15.27)	14.46 (13.40)	
vitamin B12 (pmol/L)	mean value (SD)	258 (137)	230 (158)	191 (100)	0.182
	median (IQR)	220 (160)	187 (118)	173 (67)	
NT-pro BNP (pmol/L)	mean value (SD)	22 (20)	145 (357)	117 (332)	0.069
	median (IQR)	13 (23)	21 (85)	38 (61)	

Statistically significant differences (*p* < 0.05) are given in bold. HDL: high-density lipoprotein cholesterol; LDL: low-density lipoprotein cholesterol; NT-pro BNP: N terminal pro b-type natriuretic peptide; IQR: interquartile range; SD: standard deviation.

**Table 3:** Inflammatory and hemostatic parameters according to SYNTAX score.

Parameters		SYNTAX Score			
		Group I (≤22) (*n* = 42)	Group II (23–32) (*n* = 20)	Group III (≥33) (*n* = 20)	*p* (Kruskal–Wallis Test)
leukocytes (10^9^/L)	mean value (SD)	6.91 (1.25)	7.35 (1.68)	7.31 (1.69)	0.544
	median (IQR)	6.87 (1.83)	7.06 (2.04)	7.38 (2.62)	
ESR (mm/h)	mean value (SD)	24.25 (15.18)	29.25 (23.25)	32.15 (22.45)	0.453
	median (IQR)	19.50 (27.80)	18.50 (33.00)	26.00 (27.30)	
CRP (mg/L)	mean value (SD)	3.75 (4.10)	3.82 (4.86)	7.28 (5.75)	**0.017**
	median (IQR)	2.64 (4.57)	1.75 (3.55)	6.04 (8.83)	
interleukin-6 (pg/mL)	mean value (SD)	3.61 (2.05)	4.61 (2.69)	5.03 (3.06)	0.110
	median (IQR)	2.61 (2.70)	3.77 (4.51)	4.60 (3.82)	
hemostatic parameters					
Von Willebrand factor	mean value (SD)	1.16 (0.59)	1.52 (0.62)	1.49 (0.80)	**0.040**
	median (IQR)	0.97 (0.77)	1.47 (1.02)	1.68 (1.21)	
factor VIII	mean value (SD)	2.25 (0.75)	2.21 (0.53)	2.97 (0.95)	**0.007**
	median (IQR)	2.10 (0.91)	2.32 (0.88)	2.95 (1.81)	
fibrinogen (g/L)	mean value (SD)	3.53 (0.70)	3.59 (0.62)	3.93 (0.56)	**0.030**
	median (IQR)	3.35 (0.93)	3.40 (0.97)	3.80 (0.73)	
D-dimer (mg/L)	mean value (SD)	0.74 (0.71)	1.28 (1.67)	0.68 (0.53)	0.983
	median (IQR)	0.58 (0.51)	0.45 (1.11)	0.53 (0.31)	
PAI-1 (I/U)	mean value (SD)	2.08 (0.84)	2.02 (0.51)	1.92 (0.61)	0.949
	median (IQR)	1.90 (1.25)	1.95 (0.57)	2.05 (0.67)	
prothrombin time (s)	mean value (SD)	1.06 (0.13)	1.04 (0.04)	1.06 (0.19)	0.479
	median (IQR)	1.05 (0.08)	1.03 (0.09)	1.00 (0.13)	
activated thromboplastin time (s)	mean value (SD)	33.24 (6.22)	33.84 (8.62)	38.67 (18.31)	0.722
	median (IQR)	31.78 (6.78)	30.97 (4.27)	33.43 (10.05)	

Statistically significant differences (*p* < 0.05) are given in bold. ESR: erythrocyte sedimentation rate; CRP: C-reactive protein; PAI-1: plasminogen activator inhibitor-1; IQR: interquartile range; SD: standard deviation.

**Table 4:** Echocardiography and angiography parameters according to SYNTAX score.

Parameters		SYNTAX Score			
		Group I (≤22) (*n* = 42)	Group II (23–32) (*n* = 20)	Group III (≥33) (*n* = 20)	*p*
LVEF (%)	mean value (SD)	59.33 (4.11)	56.20 (7.20)	58.45 (3.71)	0.274 *
	median (IQR)	60.00 (6.25)	59.50 (8.75)	60.00 (5.00)	
End diastolic diameter (mm)	mean value (SD)	53.29 (4.57)	54.20 (3.20)	51.85 (5.57)	0.449 *
	median (IQR)	53.00 (7.00)	54.00 (3.75)	54.00 (10.50)	
End systolic diameter (mm)	mean value (SD)	33.62 (4.69)	36.15 (2.89)	33.10 (5.15)	0.060 *
	median (IQR)	34.00 (7.00)	36.00 (4.50)	33.50 (6.75)	
Number of affected coronary arteries	mean value (SD)	1.67 (0.79)	2.65 (0.59)	2.95 (0.22)	<0.001 *
	median (IQR)	2.00 (1.00)	3.00 (1.00)	3.00 (0.00)	
Number of treated coronary arteries	mean value (SD)	1.54 (1.52)	3.35 (1.67)	4.05 (1.50)	<0.001 *
	median (IQR)	1.00 (2.00)	3.00 (2.00)	4.00 (2.00)	
Left anterior descending artery	(%)	19.00 (45.24)	19.00 (95.00)	18 (90.00)	<0.001 **
Circumflex artery	(%)	12.00 (28.57)	10.00 (50.00)	10 (50.00)	0.139 **
Right coronary artery	(%)	20.00 (47.62)	16.00 (80.00)	15.00 (75.00)	0.019 **

*: *p* values obtained by the Kruskal–Wallis test; **: *p* values obtained by the Chi-square test. Statistically significant differences (*p* < 0.05) are given in bold. LVEF: left ventricle ejection fraction; IQR: interquartile range; SD: standard deviation.

**Table 5:** Likelihood ratio test for multinomial logistic regression predicting SYNTAX score categories.

Variable	χ^2^	df	*p*
age	1.416	2	0.493
factor VIII	11.007	2	**0.004**
Von Willebrand factor	4.334	2	0.115
creatinine clearance (mL/min)	5.180	2	0.075
sex	2.522	2	0.283
diabetes mellitus	0.709	2	0.702

χ^2^: Chi-square test; df: degrees of freedom; *p* values < 0.05 given in bold indicate significant differences regarding parameters between all 3 groups.

## Data Availability

The data generated in the present study may be requested from the corresponding author.
